# Viral protein X reduces the incorporation of mutagenic noncanonical rNTPs during lentivirus reverse transcription in macrophages

**DOI:** 10.1074/jbc.RA119.011466

**Published:** 2019-12-05

**Authors:** Adrian Oo, Dong-Hyun Kim, Raymond F. Schinazi, Baek Kim

**Affiliations:** ‡ Department of Pediatrics, School of Medicine, Emory University, Atlanta, Georgia 30322; § Department of Pharmacy, Kyung Hee University, Seoul 02447, South Korea; ¶ Center for Drug Discovery, Children's Healthcare of Atlanta, Atlanta, Georgia 30322

**Keywords:** human immunodeficiency virus (HIV), SAM domain and HD domain-containing protein 1 (SAMHD1), macrophage, nucleotide, lentivirus, dNTPs, HIV-1, macrophages, rNTPs, Vpx

## Abstract

Unlike activated CD4+ T cells, nondividing macrophages have an extremely small dNTP pool, which restricts HIV-1 reverse transcription. However, rNTPs are equally abundant in both of these cell types and reach much higher concentrations than dNTPs. The greater difference in concentration between dNTPs and rNTPs in macrophages results in frequent misincorporation of noncanonical rNTPs during HIV-1 reverse transcription. Here, we tested whether the highly abundant SAM domain– and HD domain–containing protein 1 (SAMHD1) deoxynucleoside triphosphorylase in macrophages is responsible for frequent rNTP incorporation during HIV-1 reverse transcription. We also assessed whether Vpx (viral protein X), an accessory protein of HIV-2 and some simian immunodeficiency virus strains that targets SAMHD1 for proteolytic degradation, can counteract the rNTP incorporation. Results from biochemical simulation of HIV-1 reverse transcriptase–mediated DNA synthesis confirmed that rNTP incorporation is reduced under Vpx-mediated dNTP elevation. Using HIV-1 vector, we further demonstrated that dNTP pool elevation by Vpx or deoxynucleosides in human primary monocyte-derived macrophages reduces noncanonical rNTP incorporation during HIV-1 reverse transcription, an outcome similarly observed with the infectious HIV-1 89.6 strain. Furthermore, the simian immunodeficiency virus mac239 strain, encoding Vpx, displayed a much lower level of rNTP incorporation than its ΔVpx mutant in macrophages. Finally, the amount of rNMPs incorporated in HIV-1 proviral DNAs remained unchanged for ∼2 weeks in macrophages. These findings suggest that noncanonical rNTP incorporation is regulated by SAMHD1 in macrophages, whereas rNMPs incorporated in HIV-1 proviral DNA remain unrepaired. This suggests a potential long-term DNA damage impact of SAMHD1-mediated rNTP incorporation in macrophages.

## Introduction

Activated/dividing CD4+ T cells and terminally differentiated/nondividing myeloid-derived macrophages are the primary targets for type 1 and 2 human immunodeficiency virus (HIV-1 and HIV-2), as well as simian immunodeficiency virus (SIV)^2^ infections (1, 2). Interestingly, upon infection with HIV-1, both cell types display opposite cellular and virological phenotypes. Unlike infected CD4+ T cells, which undergo rapid cell death, myeloid cells display long-living phenotype following HIV-1 infections (3–5). In addition to that, HIV-1 exhibits more rapid replication kinetics in activated CD4+ T cells compared with that of nondividing myeloid cells (6–8). We have previously reported that the extremely low dNTP concentration found in macrophages (20–40 nm) kinetically restricts HIV-1 reverse transcription, which generally utilizes cellular dNTPs, whereas HIV-1 replicates at higher rate within the higher cellular dNTP pool (1–5 μm) found in activated CD4+ T cells (9). Separately, we have also demonstrated that the extremely low dNTP concentration in macrophages are due to the dNTP triphosphohydrolase (dNTPase) activities of a myeloid-specific host HIV-1 restriction factor, the SAMHD1 (SAM domain and HD domain containing protein 1) (10, 11). However, HIV-2 and some SIV strains are able to replicate rapidly even in macrophages because of their Vpx (viral protein X), an accessory protein that is not expressed by HIV-1. Indeed, Vpx elevates dNTP pool in macrophages (11) by targeting SAMHD1 for proteosomal degradation (12, 13).

Unlike dNTPs, which are utilized exclusively for DNA synthesis, cellular rNTPs are consumed for various cellular events such as RNR synthesis, as well as functioning as energy carriers and substrates of cellular kinases. As a result of the close proximity in chemical structures and large concentration discrepancy between rNTPs (millimolar range) and dNTPs (micromolar range), cellular DNA polymerases constantly misincorporate noncanonical rNTPs during synthesis of new DNA strands (14). These are common occurrences in living cells despite the presence of cellular DNA polymerases' steric gates, which consist of residues near their active sites that sterically clash with the 2′ OH group on rNTPs, hence limiting rNTP incorporation (15). Furthermore, the noncanonical rNTP misincorporation is an important contributing factor of cellular mutagenesis because the incorporated rNMP in dsDNA induces DNA polymerases pausing, which is known to be an error-prone event (14, 16, 17). In fact, although rNTP incorporation during DNA synthesis is a highly common cellular DNA damage event (16), most of living cells are capable of repairing the rNMPs incorporated in their dsDNAs. The key enzyme involved in the ribonucleotide repair is RNaseH2, which cleaves the 5′ end of a rNMP in dsDNA and initiates the excision repair mechanism known as ribonucleotide excision repair (RER) (18, 19). Interestingly, mutations in any one of the three RNaseH2 subunit genes (A, B, and C) induce the development of Aicardi–Goutieres syndrome, a rare genetic neuroimmune disorder also caused by SAMHD1 mutations (20, 21), and are characterized by hyperinterferon responses that affect brain development. It has been postulated that defects in these nucleic acid metabolism enzymes may activate the interferon-mediated innate immune systems even in the absence of any infection (22).

We previously demonstrated that HIV-1 frequently incorporates noncanonical rNTPs during viral reverse transcription in macrophages, but not in activated CD4+ T cells (23) because of a greater concentration discrepancy between rNTPs and dNTPs in macrophages compared with its actively dividing counterpart (24). Basically, an abundant dNTP pool in activated CD4+ T cells minimizes rNTP incorporation by HIV-1 reverse transcriptase (RT) during virus replication (23). Furthermore, we have also reported in the past that HIV-1 RT pauses near the rNMP incorporation sites during *in vitro* DNA synthesis (25). In this study, we tested whether SAMHD1-mediated dNTP depletion is responsible for the frequent incorporation of the noncanonical rNTPs during HIV-1 reverse transcription in macrophages and whether viral protein (X), an accessory protein expressed by HIV-2 and some SIV strains, can reduce rNTP incorporation by the virus RT via its SAMHD1-counteracting activity.

## Results

### Comparison between Vpx or dNs treatment effects on dNTP pool and HIV-1 GFP vector transduction efficiency in human primary macrophages

**Figure 1.**
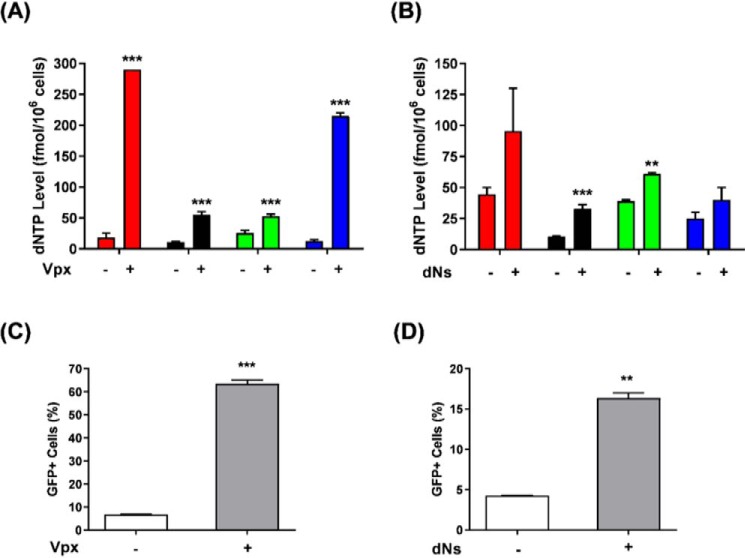
**Effects of Vpx or dNs pretreatment in macrophages.** Primary human MDMs were pretreated with VLP (Vpx −), VLP (Vpx +), or dNs for 12 or 4 h, respectively, prior to transduction with D3HIV-1 GFP vector. The nontransduced vector particles were washed after 12 h and replaced with fresh medium before a 3-day incubation at 37 °C ensued. The cells were then washed and collected for dNTP measurement: dATP (*red*), dCTP (*black*), dGTP (*green*), dTTP (*blue*) (*A* and *B*), or FACS analysis for the transduced GFP-expressing cells (*C* and *D*). The data are the means of three independent experiments, and standard deviations from the means are represented by the *error bars*.

To investigate the effect of cellular dNTP levels on noncanonical rNTP incorporation during HIV-1 reverse transcription in nondividing macrophages, we employed two different treatments that elevate cellular dNTP levels in human primary monocyte-derived macrophages (MDMs) pooled from four healthy donors: 1) virus-like particles (VLPs) containing Vpx or 2) deoxynucleosides (dNs). First, we measured the dNTP levels in primary MDMs treated with Vpx (+) VLPs or dNs by using our RT-based dNTP assay (9). As shown in Fig. 1 (*A*, Vpx, and *B*, dNs), the Vpx treatment was able to elevate dNTP levels in MDMs more effectively compared with that of the dN treatment. The less effective dNTP pool elevation observed in dN-treated MDMs can be explained by the presence of SAMHD1 in these cells, which would hydrolyze dNTPs newly synthesized from the added dNs, unlike the complete SAMHD1 degradation observed in the Vpx-treated macrophages (Fig. S1). Next, we investigated the effects of the Vpx- and dNs-mediated dNTP elevations on HIV-1 vector transduction efficiencies in MDMs. For this, we pretreated MDMs with VLP containing Vpx (Vpx +) for 12 h or dNs for 4 h prior to transduction with the D3HIV-1 GFP vector. Indeed, we observed that both Vpx (Fig. 1*C*) and dNs (Fig. 1*D*) treatments enhanced HIV-1 vector transduction efficiencies in primary MDMs represented as GFP-expressing cells when analyzed by FACS. However, HIV-1 vector transduction efficiencies were more effectively augmented by Vpx treatment than that of dNs, hence implying the effects of varying dNTP pool elevation on HIV-1 infections in MDMs.

### Biochemical simulation of rNTP incorporation during HIV-1 reverse transcription in nucleotide pools of macrophages treated with Vpx (−) or (+) VLPs

**Figure 2.**
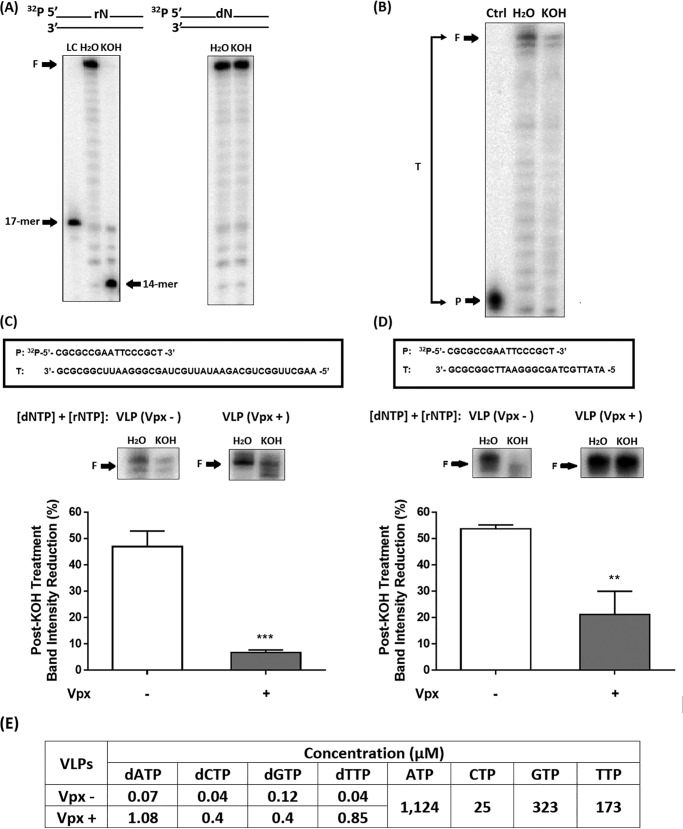
**Biochemical simulation of rNTP incorporation during HIV-1 reverse transcription in nucleotide pools of primary human MDMs treated with VLPs (Vpx − or Vpx +).** *A*, dsDNA substrate containing either a single ribonucleotide or the actual deoxyribonucleotide on one of its two 30-nucleotide-long strands, which was also the ^32^P-labeled 5′ end, was treated with 300 mm KOH (55 °C for 2 h). The resulting 14-nucleotide alkaline hydrolysis product as illustrated by the *arrow* was visualized using the phosphorus imaging device after urea–PAGE analysis. A 5′ end ^32^P-labeled 17-mer DNA was used a size loading control, LC. *B*, ^32^P-labeled 18-mer DNA primer (*P* in control (*Ctrl*)) annealed to a 40-mer RNA template (see sequences in *C*) was extended by HIV-1 RT for 20 min in the presence of dNTP/rNTP mixture pool of primary human MDMs treated with VLP (Vpx −). RT reaction products were treated with either 300 mm KOH or H_2_O and separated by urea–PAGE. The amount of full-length HIV-1 RT product (*F*) formation for each treatment group was normalized to the total radiation signal (*T*) quantified for respective lanes, and the normalized full-length product amounts were used for calculating the percentage of reduction of the full-length product by KOH caused by the rNTP incorporation. *C* and *D*, the ^32^P-labeled 5′ end 18-mer primer annealed to a 40-mer template (*C*) or 26-mer template (*D*) was extended by HIV-1 RT in the presence of dNTP/rNTP mixture pool of primary human MDMs treated with either VLPs VPx (−) or (+), and the resulting full-length products generated were magnified. Percentages of reduction in the normalized full-length RT products by KOH treatment were calculated compared with the H_2_O-treated samples. The data are the means of three independent experiments, and standard deviations from the means are represented by the *error bars. E*, concentrations of dNTPs and rNTPs used in the primer extension assays simulating respective conditions of VLPs treatments of primary human MDMs.

Next, to determine the nature of rNTP incorporation during HIV-1 reverse transcription at varying dNTP levels in MDMs, we have biochemically simulated rNTP incorporation by HIV-1 RT in the presence of dNTP concentrations found in MDMs treated with either VLPs with or without Vpx. The rNTPs incorporated in the enzymatically synthesized RT products were monitored by KOH treatment that specifically cleaves the 3′ end of any incorporated rNMP. As shown in Fig. 2*A*, when a 30-bp-long dsDNA encoding a single rNMP at the 14th nucleotide position from the ^32^P-labeled 5′ end of one strand of the dsDNA was treated with KOH, we observed the 14-mer cleavage product (see *arrow*) with little remaining full-length 30-bp DNA product (*F* in Fig. 2*A*). Conversely, there was no reduction of the 30-bp full-length product following KOH treatment of the dsDNA template without a single rNMP, hence confirming the specific rNMP cleavage in a dsDNA by the KOH treatments under our experimental conditions.

In separate experimental setups, we extended a 5′ end ^32^P-labeled 18-mer primer (P) annealed to a 40-mer template (T) by purified HIV-1 RT protein under rNTP (24) and dNTP concentrations (Fig. 1*A*) found in VLP Vpx (−) or (+) treated MDMs (see the P/T sequences in Fig. 2*C*), before the extended products were treated with KOH. As shown in Fig. 2*B*, the presence of rNTPs, which have been incorporated during HIV-1 RT-mediated DNA synthesis, was shown as reduction in the amount of full-length product generated following KOH treatment. We then compared the KOH-mediated reduction of full-length 40-bp DNA products generated by HIV-1 RT under the Vpx (−) or (+) condition. As shown in Fig. 2*C*, full-length DNA products generated under the dNTP/rNTP levels observed in the Vpx (+)–treated MDMs were less affected by the alkaline cleavage activity, whereas more than 40% of the RT product contained rNMPs, which were cleaved by KOH.

We have also employed an additional T/P with shorter and different sequences to generate the 28-bp full-length product that was treated with KOH. As shown in Fig. 2*D*, similar with the findings obtained using the longer P/T, we observed a minimal KOH-mediated reduction of the 26-bp full-length product generated at the dNTP/rNTP concentrations found in the Vpx (+) treated MDMs. The dNTP and rNTP concentrations used in these biochemical simulations were shown in Fig. 2*E*. Overall, these biochemical simulation data suggest that misincorporation of noncanonical rNTPs during HIV-1 reverse transcription in macrophages can be minimized by the dNTP-elevating effects of Vpx.

### Incorporation of rNTPs during HIV-1 reverse transcription in macrophages with elevated dNTP pool

**Figure 3.**
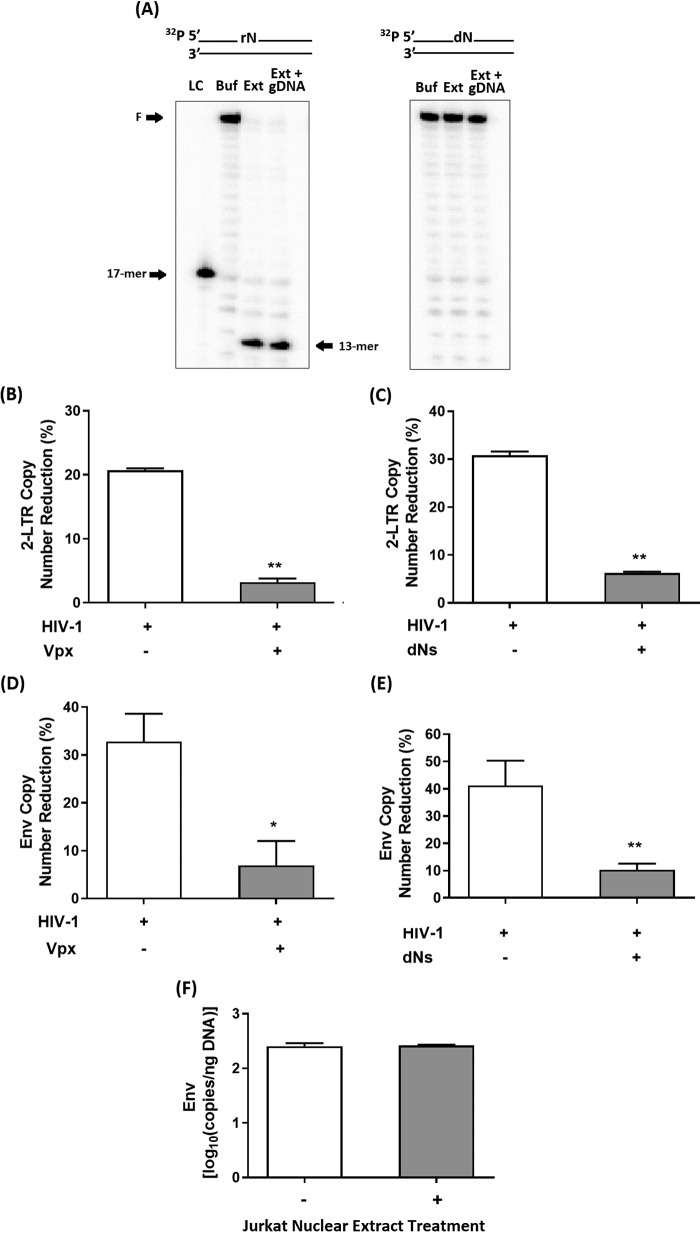
**Measurement of rNTP incorporation by HIV-1 during reverse transcription in macrophages.** *A*, dsDNA substrate containing either a single rNMP or the actual dNMP was treated with buffer (*Buf*) or Jurkat cell nuclear extract (*Ext*) containing RNaseH2 in the presence or absence of MDMs genomic DNA (*gDNA*, 200 ng) for 1 h at 37 °C. The RNaseH2 cleavage activity was visualized as a 13-nucleotide product as illustrated by the *arrow* using the phosphorus imaging device after denaturing urea–PAGE analysis. A ^32^P-labeled 17-mer DNA oligonucleotide was used a size loading control (*LC*). Primary human MDMs were pretreated with VLP (Vpx −), VLP (Vpx +), or dNs for 12 or 4 h, respectively, before they were transduced with D3HIV-1 GFP vector (*B* and *C*) or infected with infectious dual tropic HIV-1 89.6 strain (*D* and *E*). The remaining nontransduced or uninfected vector or virus particles were washed after 12 and 9 h, respectively, and fresh medium was added to the cells. Total cellular DNA was extracted at 3 days post-transduction (D3HIV-1 GFP vector) or 5 days postinfection (89.6 strain), and the extracted DNAs (200 ng) were treated with Jurkat cell nuclear extract or buffer for 1 h at 37 °C. The copy numbers of the viral genomic sequences (2LTR circle DNAs for D3HIV-1 GFP vector or *env* gene for infectious 89.6) were then measured using specific primers by qRT-PCR. *F*, human primary activated CD4+ T cells prepared from four healthy donors were infected with HIV-1 89.6 strain for 9 h before the cells were spun down and resuspended in fresh medium. After 48 h of incubation at 37 °C, total cellular DNAs isolated from the cells (200 ng) were treated with Jurkat cell nuclear extract or buffer for 1 h at 37 °C prior to HIV-1 *env* gene quantification by qRT-PCR. The data are the means of three independent experiments, and standard deviations from the means are represented by the *error bars*.

Kennedy *et al.* (23) has previously reported that rNTPs were being frequently incorporated into the HIV-1 proviral DNA during reverse transcription within the low dNTP environment of primary human MDMs, whereas the incorporation of the noncanonical molecules was almost nonexistent within the high dNTP pool of activated CD4+ T cells. In our present study, we investigated the rNTP incorporation levels by HIV-1 when we induced dNTP pool elevation in MDMs either by Vpx or dNs. The levels of incorporated rNTPs within the HIV-1 proviral DNAs were measured by the reduction of HIV-1 2LTR circle DNA copy numbers following treatments with Jurkat cell nuclear extract, which contains RNaseH2 that specifically cleaves the 5′ end of incorporated rNMPs (23). First, we tested and confirmed the RNasH2 cleavage activity of our Jurkat cell nuclear extract using a similar dsDNA template used in the earlier described KOH hydrolysis assay (Fig. 2*A*). As shown in Fig. 3*A*, relatively similar RNaseH2 cleavage products were detected in reactions conducted with or without genomic DNA isolated from MDMs (200 ng), indicating that the RNaseH2 enzyme activity was not hindered by genomic DNA present under this condition. The specificity of RNaseH2 activity in the Jurkat cell nuclear extract toward a rNMP was confirmed because no cleavage product was evident in the same dsDNA template without a single rNMP (Fig. 3*A*). These data confirmed that the RNaseH2 activity of the Jurkat cell nuclear extract effectively recognizes and cleaves the rNMPs encoded in the target DNAs under our reaction conditions.

Next, we treated MDMs with VLP Vpx (−) or (+) for 12 h before the cells were transduced with D3HIV-1 GFP vector. Following a 3-day incubation at 37 °C, the total cellular DNAs (200 ng) that also harbor HIV-1 2LTR circle DNAs were isolated from these MDMs and were treated with Jurkat cell nuclear extract or buffer. The 2LTR circle DNA copy numbers in the extracted nucleic acids were measured by quantitative RT-PCR (qRT-PCR) as previously described (23). Buffer-treated DNA served as a control that determined the 2LTR circle DNA copy numbers prior to Jurkat cell nuclear extract treatments. As demonstrated in our earlier described biochemical simulation experiment (Fig. 2, *C* and *D*), rNMP cleavage by KOH within the target dsDNAs resulted in reduced amounts of full-length DNA products generated. Hence, the cleavage of rNMPs encoded in HIV-1 2LTR circle DNAs by the RNaseH2 enzyme found in Jurkat cell nuclear extract would reduce the amount of the target gene quantified by our qRT-PCR setup. As depicted in Fig. 3*B*, we observed 20.7% reduction in 2LTR DNA copy numbers following RNaseH2 treatments in samples isolated from MDMs pretreated with VLP Vpx (−), whereas only a minimal reduction (3.2%) was observed in MDMs pretreated with VLP Vpx (+). We also observed that RNaseH2 only minimally reduced 2LTR circle copy numbers in the total DNA samples extracted from MDMs pretreated with dNs (6.2%), compared with that of untreated MDMs (30.8%) (Fig. 3*C*).

Next, we tested whether Vpx and dNs could also reduce the incorporation of the noncanonical rNTPs during reverse transcription of the infectious dual-tropic 89.6 strain in MDMs. Similarly, total cellular DNAs, which also made up HIV-1 *env* gene DNA, were extracted at the end of a 5-day incubation period at 37 °C. When the *env* gene DNA copy numbers were measured, we also observed fewer rNTPs being incorporated into HIV-1 proviral DNA during 89.6 infections in MDMs, which have been pretreated with either Vpx or dNs relative to the controls (Fig. 3*D* and 3*E*). Collectively, these findings suggest that the dNTP pool elevation in macrophages by Vpx or dNs restrict the incorporation of noncanonical rNTPs during viral replication. Similarly, minimal to no rNTP incorporation was observed in the HIV-1 *env* gene as quantified by qRT-PCR, within the high dNTP pool of activated CD4+ T cells infected with the infectious 89.6 strain (Fig. 3*F*).

### Incorporation of rNTPs by Vpx-encoding SIVmac239 and its ΔVpx mutant in macrophage

**Figure 4.**
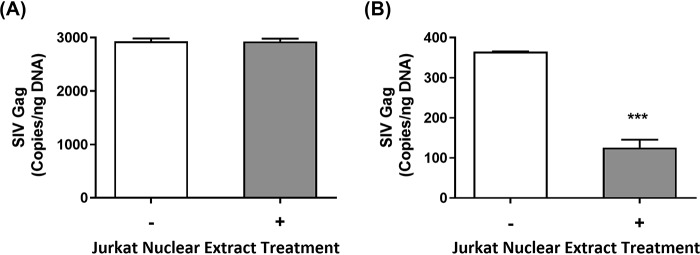
**Measurement of rNTP incorporation by SIVmac239 during reverse transcription in macrophages.** Primary human MDMs were infected with SIVmac239 (*A*) or ΔVpx SIVmac239 (*B*) mutant for 9 h before the remaining virus particles in the extracellular medium were washed and replaced with fresh medium in each well. After 5 days postinfection, total cellular DNAs were extracted, and the extracted DNAs (200 ng) were subjected to Jurkat cell nuclear extract or buffer treatment for 1 h at 37 °C before the virus *gag* gene copy numbers were measured by qRT-PCR. The data are the means of three independent experiments, and standard deviations from the means are represented by the *error bars*.

Because Vpx was capable of limiting rNTP incorporation into HIV-1 proviral DNA by modulating the cellular dNTP pool in MDMs, we then investigated the rNTP incorporation activity by SIVmac239 WT strain, which encodes Vpx compared with the ΔVpx SIVmac239 mutant. We predicted that because Vpx expressed by SIVmac239 WT is capable of degrading cellular SAMHD1 (Fig. S1), the resulting hike in dNTP levels would render the virus RT to incorporate rNTPs less frequently as opposed to the Vpx-deficient mutant. To test this prediction, we employed the RNaseH2/Jurkat cell nuclear extract-based qRT-PCR assay for determining the copy numbers of the SIVmac239 *gag* gene. MDMs were infected with SIVmac239 or its ΔVpx mutant for 5 days before total DNAs from the infected cells were harvested for subsequent quantification. As shown in Fig. 4*A*, the total DNA samples extracted from MDMs infected with SIVmac239 displayed only minimal reduction of the *gag* gene copy number following treatment with Jurkat cell nuclear extract. However, we observed a significant reduction of the *gag* gene copy number in the samples extracted from MDMs infected with the ΔVpx SIVmac239 mutant (Fig. 4*B*). This observation further supports that Vpx is capable of restricting rNTP incorporation during viral replication by modulating cellular dNTP pool in infected macrophages. In addition, the observed discrepancy in rNTP incorporation between SIVmac239 (Fig. 4) and HIV-1 (Fig. 3) in macrophages results from their different ability to counteract host SAMHD1 dNTPase and to elevate cellular dNTP levels in macrophages.

### RNaseH2-induced ribonucleotide excision repair mechanism in macrophages

**Figure 5.**
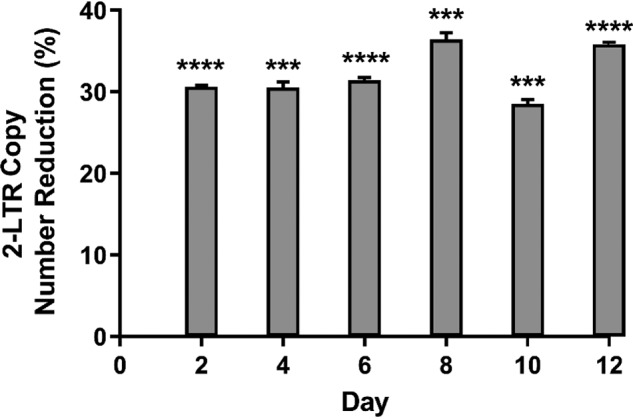
**Ribonucleotide excision repair profile on rNMPs incorporated in HIV-1 proviral DNAs in macrophages.** Primary human MDMs were transduced with D3HIV-1 GFP vector for 12 h, and the cells were washed and replaced with fresh medium. On specific days postinfection (2, 4, 6, 8, 10, and 12 days), total cellular DNAs were extracted, and the extracted DNAs (200 ng) were treated with Jurkat cell nuclear extract or buffer for 1 h at 37 °C. The treated DNA samples were subsequently quantified for HIV-1 2LTR circle DNA using qRT-PCR, and the percentages of reductions of the 2LTR circle DNA copy number by the Jurkat cell nuclear extract treatment were plotted. The data are the means of three independent experiments, and standard deviations from the means are represented by the *error bars*.

Because incorporated rNTPs are detrimental toward regular DNA replication, removal of the embedded ribonucleotides are initiated by the RNaseH2 enzyme via a DNA damage repair mechanism known as the RER pathway (18, 19). The level of RNaseH2 protein and its enzymatic activity were relatively lower in MDMs as compared with replicating cell lines such as the activated CD4+ T cells, as published previously (23). Hence, we investigated the long-term fates of incorporated rNMPs incorporated in HIV-1 proviral DNAs within infected MDMs. First, MDMs were transduced with D3-HIV1 GFP vector, and total cellular DNAs were extracted from the infected cells at different time points of infection (2, 4, 6, 8, 10, and 12 days postinfection). Then we monitored the changes in HIV-1 2LTR circle copy numbers in the total DNAs extracted from the infected MDMs post-RNaseH2/Jurkat cell nuclear extract treatments. As shown in Fig. 5, we observed that there were no significant changes in reduction of HIV-1 2LTR DNA copy numbers following Jurkat cell nuclear extract treatments across the 12 days of infection. This suggests that a majority of the rNMPs incorporated in HIV-1 proviral DNAs remain unrepaired even up to 12 days in macrophages.

## Discussion

Although the daily rate of DNA damage was estimated to be as high as 50,000 lesions in every human cell (26), rNTPs that are incorporated at a frequency of 2 ribonucleotides per kb of human genome by DNA polymerases serve as the most common source of DNA lesions (18). Misincorporation of rNTPs can be commonly observed across all levels of living organisms, from the single-celled prokaryotes to the highly complex systems of eukaryotes. The polymerase III of *Escherichia coli* was predicted to incorporate ∼2,000 ribonucleotides into its 4.6 Mb chromosome (27), whereas yeast's 12.5 Mb genome was estimated to contain 10,000 of the noncanonical molecules (14). In fact, we have previously estimated that HIV-1 RT incorporates 1 ribonucleotide in every 146 nucleotides during reverse transcription within the low dNTP pool of macrophages (23).

In our current study described here, we have clearly shown that within an elevated dNTP pool environment, rNTP incorporation by HIV-1 RT was effectively suppressed (Figs. 2 and 3). This proves that in the presence of high dNTP pool, HIV-1 RT exhibits a higher tendency to incorporate dNTPs as their substrates for DNA synthesis, a similar pattern also observed in activated CD4+ T cells as reported in our previous study (23). The incorporation of canonical dNTPs during HIV-1 reverse transcription are especially crucial in macrophages that contain relatively lower amounts of the RNaseH2 repair enzyme compared with the actively dividing CD4+ T cells (23). RNaseH2 plays a central role in the RER pathway by recognizing and cleaving the 5′ end of rNMPs found in DNA strands (18). This resulted in the formation of flaps that would eventually be removed by nucleases such as Exo1 or FEN1, before the repaired DNA strands are sealed by ligases. Therefore, missing any of these multiple RER players will result in a lack of an efficient RER capacity, leading to sustained accumulation of the noncanonical rNMPs in replicating DNA molecules, as observed in the HIV-1 genome following a long period of infection in macrophages (Fig. 5).

The detrimental effect of rNTP incorporation into replicating DNA strand was evident when McElhinny *et al.* (14) observed the pausing effect in DNA polymerase acting on DNA template containing a single rNMP. The hydrolysis of the phosphate bonds of incorporated rNTPs result in the formation of nicks that stall the replication fork, hence generating pause sites near the base location at which rNMP(s) is/are present. Utilizing a biochemical approach, we have previously observed similar pausing effects during HIV-1 reverse transcription within low dNTP pool of macrophages, when the DNA template contained a single incorporated rNMP (25). Pause sites serve as potential sources of mutagenesis because of the switching from high-fidelity to low-fidelity polymerases when respective replisomes are stalled at the incorrect/damaged base(s) (28). In addition, even though the base of a rNMP may exhibit the usual Watson–Crick potential, the conversion from its B-form to its A-form has been shown to result in restricted extensions during DNA synthesis (29). Rearrangement of difficult-to-extend sites into misaligned intermediates would eventually generate mutagenic nucleotide(s) insertion or deletion during subsequent extension of the replicating DNA strands (30). Hence, active incorporation of rNTPs by HIV-1 RT during virus replication, especially in the low dNTP environment of macrophages, could possibly result in altered viral genomes and replication kinetics. The resulting viral mutagenesis and diversity would promote HIV-1 to escape from host immune selection, as well as anti-viral drug treatments. Furthermore, the characterization of the rNTP incorporation “hot” and “cold” spots throughout the viral genome by using several reported methods (31, 32) would be able to inform any sequence-specific preference of the RT-mediated rNTP incorporation.

The role of Vpx in limiting rNTP incorporation by lentiviral RTs was further proven by the active incorporation of rNTPs during reverse transcription of Vpx-deficient SIV mutant in macrophages (Fig. 4) and the biochemical failure of rNTP incorporation by SIVmac239 RT at high dNTP concentrations found in macrophages treated with Vpx (Fig. S2). Collectively, this study reveals that the possibly mutagenic, noncanonical rNTP incorporation during HIV-1 reverse transcription in macrophages can be counteracted by lentiviral Vpx, which enhances cellular dNTP levels via its SAMHD1 degradation activity.

## Experimental procedures

### Cells

Primary human monocytes were isolated and pooled from buffy coats of four healthy blood donors (New York Blood Service, Long Island, NY) by positive selection using MACS CD14 microbeads (Miltenyi Biotec) as described in our previous study (33). The monocytes were differentiated into MDMs in the presence of 5 ng/ml human granulocyte-macrophage colony-stimulating factor (Miltenyi Biotec) for 7 days prior to respective experiments.

### Virus-like particles

VLPs (Vpx − or Vpx +) were prepared as described previously (34). Briefly, supernatants were collected on days 2 and 3 post-transfection from 293 FT cells transfected with 40 μg of pVpx − VLP or pVpx + VLP (generously provided by Dr. Florence Margottin-Goguet and Dr. Nathaniel Landau) and 20 μg of pVSV-g in the presence of polyethylenimine (1 mg/ml). Following centrifugation at 1,200 rpm for 7 min to remove cellular debris, the supernatants were overlaid above a 25% (w/v) sucrose solution before the VLPs were concentrated by ultracentrifugation (SW28 rotor) at 22,000 rpm for 2 h. The pellets obtained were resuspended in Hanks' balanced salt solution and aliquoted into smaller volumes. The aliquots were flash-frozen using ethanol before being stored at −80 °C until needed for the experiments.

### HIV-1 GFP vector and infectious viruses

The *env*-deficient HIV-1 NL4–3 based D3HIV-1 GFP vector, which encodes the entire HIV genes except *env* gene and *nef* gene replaced with eGFP gene, was generated by transfecting 293 FT cells with pD3-HIV (40 μg) and pVSV-g (20 μg) plasmids using 1 mg/ml of polyethylenimine as described in the past (35). Supernatants collected on days 2 and 3 post-transfection were centrifuged at 1,200 rpm for 7 min to remove unwanted debris before being subjected to ultracentrifugation (SW28 rotor) at 22,000 rpm for 2 h. The concentrated D3HIV-1 vector was subsequently aliquoted into smaller volumes and stored in −80 °C until needed. ELISA (Advanced BioScience Laboratories Inc.) was performed to quantitate p24 antigen, whereas GFP expression levels were determined by fluorescence microscopic observation following transduction of 293 FT cells with the vector for 2 and 3 days.

Infectious HIV-1 89.6, SIVmac239, and ΔVpx SIVmac239 were collected from the supernatant of 293 FT cells transfected with respective virus molecular clone plasmids: HIV-89.6 (catalog no.1966), SIVmac239 (catalog no.12249), and ΔVpx SIVmac239 (catalog no.12252) (National Institutes of Health AIDS Reagent Program, Division of AIDS, NIAID, National Institutes of Health). The resulting viruses were propagated in CEMx174 cells for 10 passages until the β-lactamase gene encoded in the plasmid was not detected by PCR to confirm the absence of the 89.6 plasmid contamination. HIV-1 p24 an SIV p27 levels were constantly monitored and quantified on every passage using ELISA (Advanced Bioscience Laboratories Inc.). Virus stocks were aliquoted into tubes and stored at −80 °C for future usage in respective experiments.

### HIV-1 RT-based dNTP measurement (9)

Using cold 60% methanol, MDMs were lysed. After heating the lysate at 95 °C for 3 min, any unwanted debris was removed by centrifugation at 14,000 rpm. The supernatants were dried using a SpeedVac for 2 h, and the resulting pellets obtained were dissolved in 20 μl of water. Using 2 μL of the resuspended sample, a primer extension assay by HIV-1 RT involving 5′ ^32^P-labelled primer (5′-GTCCCTCTTCGGGCGCCA-3′) annealed to any one of the four different templates (3′-CAGGGAGAAGCCCGCGGTN-5′) was conducted, where *N* is either one of the DNA nucleobases as described previously (10). The reaction products were terminated with 10 μl of 40 mm EDTA, 99% formamide and denatured at 95 °C for 5 min. 4 μl of the resulting reaction mixture was resolved on 14% polyacrylamide–urea denaturing gels (SequaGel, National Diagnostics). Gel images were captured using a phosphorus imaging device (Bio-Rad), and respective dNTP levels were analyzed using the Quantity One software (Bio-Rad).

### Alkaline hydrolysis assay

To verify KOH's cleavage activity toward the 3′ end of ribonucleotides (Fig. 2*A*), dsDNA templates made up of either a single ribonucleotide (5′-GCACAATATTGCT**rA**GCGGGAATTCGGCGCG-3′) or the actual deoxyribonucleotide annealed to its complementary template were treated with 300 mm KOH for 2 h at 55 °C. The reaction product was neutralized with 300 mm HCl prior to the addition of 10 μl of 40 mm EDTA, 99% formamide. 4 μl of the final reaction mixture was loaded into 14% polyacrylamide–urea denaturing gel (SequaGel, National Diagnostics), and the resulting cleavage product was visualized using a phosphorus imaging device (Bio-Rad).

Separately, the effects of KOH hydrolysis toward HIV-1 RT reaction products formation (Fig. 2, *C* and *D*) were performed as described (23) utilizing primer templates of different lengths: ^32^P-labeled 18-mer DNA primer (5′-CGCGCCGAATTCCCGCT-3′) annealed to a 40-mer RNA template (5′-AAGCUUGGCUGCAGAAUAUUGCUAGCGGGAAUUCGGCGCG-3′; Fig. 2*C*) or similar primer as above annealed to a 26-mer DNA template (5′-AATATTGCTACGCCCAATTCG GCGCG-3′; Fig. 2*D*).

### Jurkat cell nuclear extract

Nuclear extract from Jurkat cells were prepared as described in previous studies (23, 36). Fundamentally, Jurkat cells were lysed by Dounce homogenization and centrifuged at 3,300 × *g* for 15 min. The separated nuclear fraction was subjected to extraction on ice using low-salt (0.02 m KCl) and high-salt (1.6 m KCl) buffers. The extracts were centrifuged at 22,605 × *g* for 30 min, and the resulting nuclear extract was dialyzed in dialysis buffer without 0.5 m DTT overnight at 4 °C. The extract was aliquoted into smaller volumes and stored in −80 °C.

### RNaseH2 activity assay

Jurkat cell nuclear extract prepared earlier was tested for its RNaseH2 activity as described (23). In a reaction consisting of 10 nm dsDNA substrates similarly utilized in the above-mentioned KOH activity verification assay, 20 μm oligo(dT)_20_ and 1× ThermoPol reaction buffer (New England Biolabs), 8 μl of the extract was added and incubated at 37 °C for 30 min. Separately, genomic DNA isolated from 293 FT cells was added into the reaction mixture to evaluate RNaseH2 activity of the extract in the presence of genomic DNA. The reactions were terminated with 10 μl of 40 mm EDTA, 99% formamide, and the reaction products were denatured at 95 °C for 5 min. Using 14% polyacrylamide–urea denaturing gel (SequaGel, National Diagnostics), 4 μl of the final reaction mixture was separated, and the resulting RNaseH2 cleavage product was visualized and analyzed using a phosphorus imaging device (Bio-Rad) and Quantity One software (Bio-Rad), respectively.

### qRT-PCR–based rNTP incorporation assay

Viral nucleic acids were extracted from MDMs transduced with D3HIV-1 GFP vector or infected with infectious HIV-1 89.6 or SIVmac239/ΔVpx SIVmac239 mutant in respective experiments using the Wizard® genomic DNA purification kit (Promega). The DNA samples were treated with Jurkat cell nuclear extract or buffer for 30 min at 37 °C. By performing qRT-PCR using primer sets and probes designed for HIV-1 2LTR circle DNA, forward and reverse primers anneal to 75 bp upstream and 33 bp downstream from 3′ end of the 5′-LTR region and 5′ end of the 3′-LTR region, respectively (10), and *env* gene: 8782–8928 (37) as well as SIV *gag* coding region: 1120–1192 (probe was modified with FAM/ZEN) (38), respective viral DNA copy numbers following treatment with Jurkat cell nuclear extract was analyzed and compared with that of buffer control. The target genes were amplified in the presence of specific forward and reverse primers (0.5 μm each), the probe (0.25 μm), and 200 ng of the isolated total cellular genomic DNA using LightCycler 480 Probes master kit (Roche). The thermal cycler parameters were set at 45 cycles of 95 °C for 10 s, 60 °C for 30 s, and 72 °C for 1 s, before the reactions were cooled for 30 s at 40 °C. The levels of rNTP incorporation into viral DNA were measured as a percentage of reduction in DNA copy numbers in Jurkat cell nuclear extract treated samples relative to the buffer control.

### Statistical analyses

All data were analyzed using GraphPad Prism (version 8) for Windows. Unpaired *t* tests were performed to determine the significance of the readings obtained from respective experimental setup relative to the mock-treated controls, including data for each time point in Fig. 5 that were analyzed independently to its buffer control. The results are presented as means ± S.E. *p* < 0.05 was represented as *; *p* < 0.01 was represented as **; *p* < 0.001 was represented as ***; and *p* < 0.0001 was represented as ****.

## Author contributions

A. O. data curation; A. O. software; A. O. formal analysis; A. O. validation; A. O. visualization; A. O. methodology; A. O., D.-H. K., R. F. S., and B. K. writing-review and editing; D.-H. K., R. F. S., and B. K. conceptualization; R. F. S. and B. K. resources; R. F. S. and B. K. funding acquisition; B. K. supervision; B. K. investigation; B. K. writing-original draft; B. K. project administration.

## Supplementary Material

Supporting Information

## Footnotes

2 The abbreviations used are:

SIV: simian immunodeficiency virus
RT: reverse transcriptase
RER: ribonucleotide excision repair
MDM: monocyte-derived macrophage
VLP: virus-like particle
dN: deoxynucleoside
P: primer
T: template
qRT-PCR: quantitative RT-PCR.
