# Supplementary material for: Viral protein X reduces the incorporation of mutagenic noncanonical rNTPs during lentivirus reverse transcription in macrophages
Source: J Biol Chem. 2019 Dec 5;295(2):657–66. doi: 10.1074/jbc.RA119.011466 (PMC6956541; doi:10.1074/jbc.RA119.011466)
Supplement: Supporting Information [file supp_295_2_657__index.html]

Viral protein X Reduces the Incorporation of Mutagenic Noncanonical rNTPs during Lentivirus Reverse Transcription in Macrophages — Vpx reduces rNTP incorporation of lentiviruses in macrophage — Viral protein X reduces the incorporation of mutagenic noncanonical rNTPs during lentivirus reverse transcription in macrophages — Vpx reduces rNTP incorporation of lentiviruses in macrophage — Supporting Information 

# Viral protein X reduces the incorporation of mutagenic noncanonical rNTPs during lentivirus reverse transcription in macrophages

## Supporting Information

- Supplementary Figures - Supplementary Figures 1 and 2
