# Supplementary material for: Viral protein X reduces the incorporation of mutagenic noncanonical rNTPs during lentivirus reverse transcription in macrophages
Source: J Biol Chem. 2019 Dec 5;295(2):657–66. doi: 10.1074/jbc.RA119.011466 (PMC6956541; doi:10.1074/jbc.RA119.011466)
Supplement: Supporting Information [file supp_RA119.011466_156442_2_supp_438769_q21kb5.pdf]

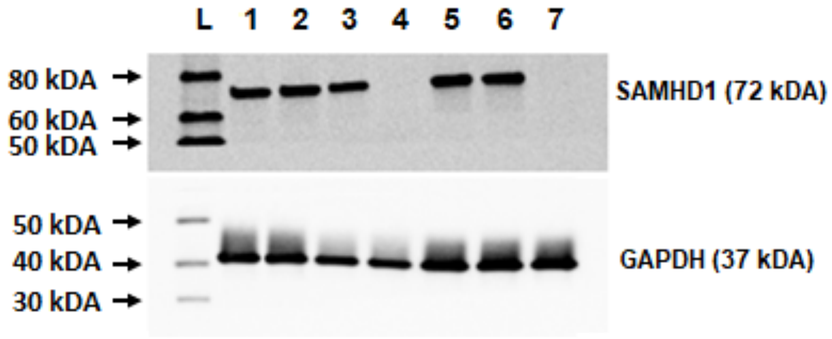

Supplementary Figure 1. SAMHD1 levels in primary human monocyte derived macrophages under varying experimental conditions employed in this study. SAMHD1 levels in macrophages with **(1)** no infection, **(2)** HIV-1 89.6 infection, **(3)** treatment with Vpx - VLPs and HIV-1 89.6 infection, **(4)** treatment with Vpx + VLPs and HIV-1 89.6 infection, **(5)** dNs treatment, **(6)** SIVmac239 ΔVpx mutant infection, and **(7)** SIVmac239 WT infection were analysed. The cells were collected post 72 h treatments/infections, and the cell lysates obtained were used for western blots using human SAMHD1 antibody. GAPDH was used as a loading control. L: Size markers (SAMHD1: 80, 70 and 60 kDA: GAPDH: 50, 40 and 30 kDA from the top of the blots).

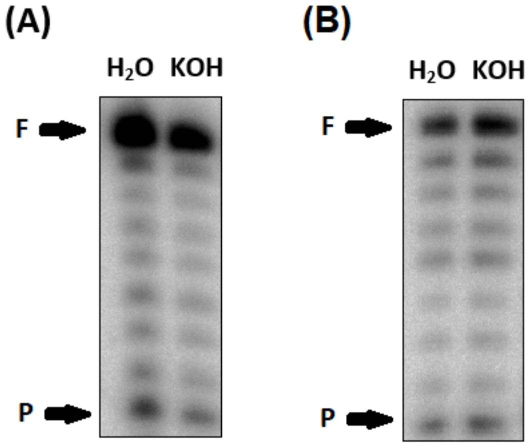

Supplementary Figure 2. Biochemical simulation of rNTPs during lentivirus reverse transcription in nucleotide pools of primary human MDMs treated with VLP (Vpx +). ^{32}P-labelled 18-mer DNA primer ("P") annealed to a 26-mer DNA template was extended by (A) HIV-1 or (B) SIV RT in the presence pf dNTP/rNTP concentrations of primary human MDMs treated with VLP (Vpx +). Full-length RT product is depicted as "F".
